# Targeting aggression with prefrontal high-definition transcranial direct current stimulation

**DOI:** 10.1038/s41598-026-39423-5

**Published:** 2026-02-10

**Authors:** Luca Lasogga, Lena Hofhansel, Chiara Gramegna, Ute Habel, David M.A. Mehler, Ruben C. Gur, Judith Dammers, Andreas Reif, Carmen Weidler

**Affiliations:** 1 Department of Psychiatry, Psychotherapy and Psychosomatics, Faculty of Medicine, RWTH Aachen, MTI1, Office 117, Wendlingweg 2, Pauwelsstraße 30, 52074 Aachen, North Rhine- Westphalia Germany; 2 Institute of Neuroscience and Medicine, Research Center Jülich, JARA- Institute Brain Structure Function Relationship (INM 10), Wilhelm-Johnen-Straße, 52438 Jülich, Germany; 3 Institute for Translational Psychiatry, University of Münster, 48149 Münster, Germany; 4 Cardiff University Brain Research Imaging Centre (CUBRIC), School of Psychology, Cardiff University, Cardiff, CF24 4HQ UK; 5 Brain Behavior Laboratories, Department of Psychiatry, Perelman School of Medicine, University of Pennsylvania, Philadelphia, USA; 6 Department of Psychiatry, Psychosomatic Medicine and Psychotherapy, University Hospital Frankfurt, 52074 Frankfurt, Germany

**Keywords:** Aggression, Provocation, High-definition tDCS, rIFG, fMRI, Neuroscience, Psychology, Psychology

## Abstract

Heightened aggression is associated with behavioural and neural deficits in inhibitory control. Transcranial direct current stimulation (tDCS) shows promise in facilitating inhibitory control and reducing aggression. However, the mixed effects and widespread electric fields of tDCS warrant more precise stimulation methods. High-definition tDCS (HD-tDCS) may enhance focality to target the right inferior frontal gyrus (rIFG), a key region in inhibitory control. In a double-blind and sham-controlled study, we investigated behavioral and neural differences between anodal and sham HD-tDCS in a subsequent Taylor Aggression Paradigm (TAP). Anodal HD-tDCS was applied at 1.5 mA for 20 min over the rIFG. Reference electrodes were located at TP8, PZ, FC3, and FP1. 41 healthy male participants were randomly assigned to either anodal (19) or sham (22) HD-tDCS and completed the TAP during functional magnetic resonance imaging. Anodal HD-tDCS did not directly reduce aggression; however, the stimulation weakened the effect of provocation on aggressive behaviour. Imaging results indicated that anodal HD-tDCS was associated with a positive provocation-related increase of bilateral parietal lobule (IPL) activation. Taken together, behavioural and neuroimaging findings suggest that anodal rIFG stimulation may engage the inhibitory control network as reflected in reduced responsiveness to provocation and increased activation during provocation.

## Introduction

Aggression imposes a substantial burden on both societal and individual well-being. Aggressive behavior can pose a serious risk to healthcare personnel in clinical settings^1^ and has therefore become an issue of growing clinical and public concern. Violent crimes are disproportionately committed by men^2^ rendering aggression in the male population a matter of particular relevance. Experimental evidence indicates that provocation, in particular, is associated with increased aggressive behavior^3–5^, potentially by eliciting a reactive form of aggression characterized by impulsive actions arising from deficits in emotion regulation and inhibitory control^6,7^. These underlying deficits may help explain why aggression is often difficult to manage, and they highlight the need for effective interventions. However, current approaches remain limited in the efficacy compared with treatments for other clinically relevant conditions, such as affective disorders^8^.

Non-invasive brain stimulation (NIBS) gained popularity in the modulation of aggression. Specifically transcranial direct current stimulation (tDCS) constitutes a promising approach for aggression-related treatments. TDCS can modulate brain activation by applying a direct current to the scalp, thereby changing the resting membrane potential of neurons below the stimulation electrode^9^. Anodal and cathodal tDCS have generally been assumed to increase and decrease cortical excitability, respectively^10^. However, this dichotomy may be less consistent in cognitive functions^11^. Studies applying tDCS over various prefrontal brain regions in an attempt to modulate aggression have yielded mixed and inconsistent findings^12–14^. This heterogeneity may be attributable to the wide range of parameters and study designs used across studies^13^. For instance, tDCS studies often vary with respect to their montage parameters, including the choice of target region and polarity of the stimulation which may give rise heterogeneous results. Previous studies suggest that tDCS over the dorsolateral prefrontal cortex (DLPFC) can influence aggression; however, although some findings converge, inconsistencies remain.

With respect to bilateral DLPFC stimulation in male participants, right-anodal/left-cathodal tDCS has been reported to show less aggressive behavior compared to sham in an alcohol-abusing population^5^, but it has also been reported to increase aggression^15^ or to have no effect in healthy individuals^16^. Similarly, left-anodal/right-cathodal tDCS over the DLPFC has been linked to reduced aggression in methamphetamine abusers^17^, but to increased aggression in healthy controls^18^. Research targeting the ventrolateral PFC (VLPFC) has shown partially inconsistent findings but overall supports the notion that tDCS targeting the right inferior frontal gyrus (rIFG) may successfully modulate aggression. Specifically, right-anodal/left-cathodal tDCS has been associated with reduced aggression^19–21^, but it has also been reported to resulted in null effects^22,23^. In contrast, left-anodal/right-cathodal tDCS has been linked to increased aggression^23^, while similarly producing null effects^22^. Additionally, unilateral anodal tDCS applied over the medial PFC, with a single cathodal electrode placed on the shoulder, has been associated with reduced anger^24^.

Although findings have been mixed, tDCS targeting the VLPFC, particularly the right inferior frontal gyrus (rIFG), appears to be a promising approach for modulating aggression^19–21^. The rIFG has been implicated in response inhibition, a core component of inhibitory control, that is crucial for the regulation of aggression^6,25–27^. The rIFG forms part of a broader neural network that includes the inferior parietal lobule (IPL), DLPFC, insula, middle cingulate gyrus, and somatosensory areas, and has been linked to inhibitory control functions^28,29^. While a growing body of tDCS research highlights involvement of the rIFG in aggression^6^ and its modulatory effects^19–21^, the spatial precision of tDCS remains limited.

Several earlier studies have utilized relatively large conventional tDCS electrodes (e.g., 5 × 7 cm), which may recruit widespread prefrontal regions. However, when targeting functionally specific subregions such as the rIFG, the resulting electric fields may tend to spread across adjacent cortical areas, limiting spatial specificity. High-definition tDCS (HD-tDCS) addresses wide electric field distributions by enabling more spatially confined stimulation and producing more focal cortical activation patterns^30^. In addition to improved spatial specificity, HD-tDCS has been associated with longer lasting plasticity-related effects compared with conventional tDCS. Previous research indicates that HD-tDCS can induce up to 30 min longer-lasting neural plasticity changes as compared to conventional tDCS^31^. To our knowledge, only one study has applied HD-tDCS to the ventromedial PFC and reported a subsequent reduction in aggression^32^, highlighting the potential importance of enhanced regional specificity for aggression modulation. Alongside promising effects of HD-tDCS on aggression, the precision of HD-tDCS makes it a promising approach for targeting functionally specific brain regions such as the rIFG.

The aim of the present study was to examine the effectiveness of HD-tDCS in modulating aggression. Building on previous evidence implicating the rIFG as a key region in aggression regulation, we targeted the rIFG using HD-tDCS to capitalize on its enhanced spatial specificity. In a double-blind, sham-controlled study, we combined functional magnetic resonance imaging (fMRI) and HD-tDCS to investigate behavioral and neural correlates of aggression. Participants underwent a single session of HD-tDCS and subsequently completed a competitive reaction time task during fMRI acquisition. We expected that participants receiving anodal HD-tDCS over the rIFG would exhibit lower levels of aggressive behavior as compared to those receiving sham stimulation. Furthermore, we expected differences in neural activation between anodal and sham conditions, primarily within the rIFG and other regions of the inhibitory control network, including the DLPFC and IPL. Ultimately, this line of research aims to inform the development of NIBS protocols with longer lasting effects that strengthen inhibitory control and reduce aggression. The present study contributes to the still limited literature on the effects of HD-tDCS on aggression.

## Results

### Demographical statistics

Group comparisons are presented in Table 1. After false detection error correction (FDR), no significant differences emerged with respect to age, education, cognitive measures and self-reported aggression and impulsivity. Comparing groups without correction revealed a significant difference between anodal and sham HD-tDCS groups in the vocabulary test score.

**Table 1:** Descriptive statistics and sample characteristics.

Variable	Sham mean (SD)	Anodal mean (SD)	t	df	*p*	FDR-cor
N	22	19				
Age	28.36 (9.1)	29.74 (10.81)	-0.44	35.41	0.665	0.915
Education	13.91 (1.48)	13.37 (2.22)	0.9	30.6	0.373	0.828
TMT_A	22.95 (6.29)	22.86 (8.42)	0.04	32.99	0.969	0.974
TMT_B	45.2 (21.87)	48.57 (22.66)	-0.48	37.7	0.632	0.915
WST	31.57 (3.28)	27.79 (7.2)	2.1	24.61	**0.046**	0.507
AQ Physical	23.05 (3.97)	24.21 (5.3)	-0.79	33.03	0.437	0.828
AQ Verbal	12.5 (3.11)	11.74 (3.28)	0.76	37.46	0.452	0.828
AQ Anger	16 (4.6)	15.95 (4.58)	0.04	38.19	0.971	0.974
AQ Hostility	15.86 (6.26)	13.79 (2.7)	1.41	29.43	0.169	0.828
RPQ total	8.68 (5.23)	7.05 (4.12)	1.11	38.7	0.272	0.828
BIS 11 total	59.86 (9.15)	59.95 (6.88)	-0.03	38.33	0.974	0.974

SD = standard deviation, t = t-value, df = Degrees of freedom, p = pvalue, FDR-cor = p-value false detection error corrected for multiple testing, TMT = trial-making test, WST = German vocabulary test, AQ = Aggressionquestionnaire, RPQ = Reactive proactive questionnaire, BIS-11 = Barratt impulsiveness scale-11, bold font = significant.

### Blinding and side effects

Side effects can be viewed in Table 2. These side effects included 12 categories assessed in previous HD-tDCS research^33^. Items were rated on a Likert-scale ranging from 1 to 4. We did not observe any severe side effects apart from tingling and itching sensations as well as sleepiness. The latter might largely be due to the additional fMRI exposure. A chi-square test indicated no association between the stimulation condition participants believed they had received and the condition they actually received, X^2^ (df = 1, 41) = 0.001, *p* = 0.967.

**Table 2:** Side effects.

Side effect	Mean	Percentage
Warming	1.707317	21.95122
Numbness	1.170732	2.439024
Pain	1.341463	7.317073
Itching	2.317073	43.90244
Burning sensation	1.731707	19.5122
Tingling	2.439024	46.34146
Metal taste	1	0
Sleepiness	2.243902	46.34146
Dizziness	1.170732	2.439024
Concentration problems	1.804878	26.82927
Nausea	1.04878	2.439024
Headache	1.097561	2,439024

Percentage refers to how many participants scored above 2 on the item.

### Behavioral results

The linear mixed model showed an estimated effect size of R^2^_conditional_ = 0.39. The findings are presented in Table 3. Two different random effects structures were compared in terms of Akaike’s information criterion (AIC), Bayesian information criterion (BIC) and explained variance. The current model performed best and was thus, carried out for the behavioral analysis.

**Table 3:** Mixed linear model fixed effects.

Variable	β	SE	Lower 95%	Upper 95%	t	*p*	df
Intercept	2.72	0.23	2.27	3.17	11.89	**< 0.001**	40.66
Outcome	-0.08	0.08	-0.23	0.07	-1.04	0.296	2986.61
HD-tDCS	-0.13	0.23	-0.57	0.31	-0.57	0.570	38.82
Provocation	0.25	0.02	0.22	0.28	16.73	**< 0.001**	2983.99
HD-tDCS x Provocation	0.06	0.02	0.03	0.08	3.67	**< 0.001**	2984.06

β = beta coefficient, SE = standard error, LB = lower bound, UB = Upper bound, p = p-value, df = degrees of freedom, bold font = significant.

The analysis revealed no significant main effect of the outcome of the previous trial, nor a significant main effect of HD-tDCS. In contrast, a significant main effect of provocation was observed, indicating that higher levels of provocation were associated with increased punishment selections. The model further revealed a significant interaction effect between HD-tDCS condition and provocation on punishment selection indicating a difference in slopes between HD-tDCS groups. Detailed post-hoc effects are shown in Table 4.

**Table 4:** Post-hoc test comparing the effect of provocation on punishment selection between anodal and Sham HD-tDCS.

Slope contrast	β_1_	SE_1_	df_1_	t-ratio	*p*_1_
Sham - Anodal	0.11	0.03	2984.27	3.66	**< 0.001**
**Slope per group**	β_2_	SE_2_	*t*	*p*_2_	
Sham	0.31	0.02	15.11	**< 0.001**	
Anodal	0.20	0.02	8.85	**< 0.001**	

β = beta coefficient, SE = standard error, p = p-value, df = degrees of freedom, bold font = significant.

**Fig. 1:**
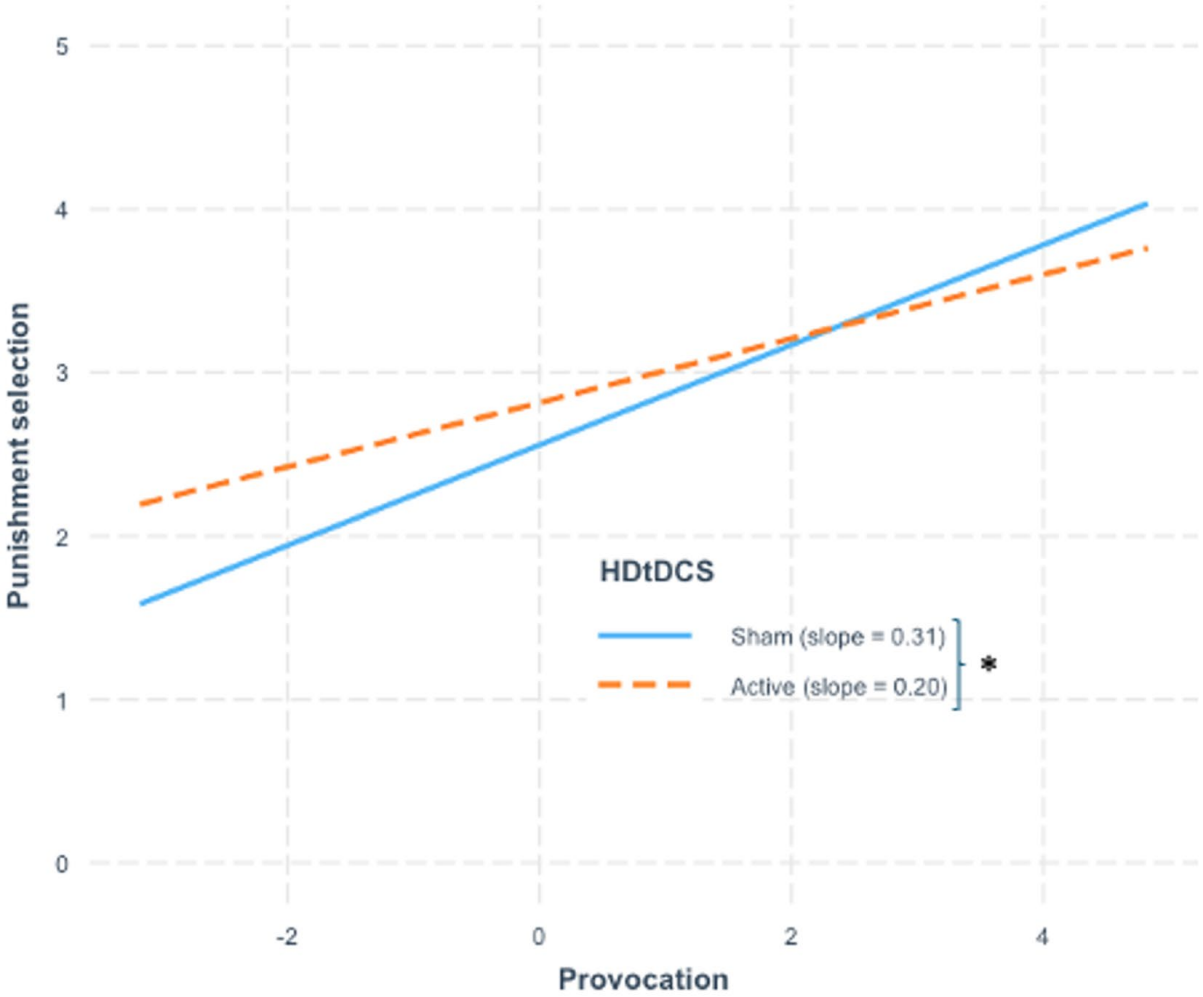
Effect of provocation on punishment selection by HD-tDCS group. Interaction between HD-tDCS condition and mean-centered provocation (x-axis) on punishment selection (y-axis). The blue line represents the association between provocation and punishment selection in the anodal HD-tDCS group, whereas the orange line represents the corresponding association in the sham group. The asterisk indicates the significant difference between slopes.

Table 4 entails the post-hoc analysis revealing a significant difference between HD-tDCS groups in the association between provocation intensity and subsequent punishment selection. Anodal HD-tDCS was associated with a shallower slope in the relationship between provocation and punishment selection compared to the sham group, which exhibited a steeper slope. The interaction is illustrated in Fig. 1.

### Imaging results

**Fig. 2:**
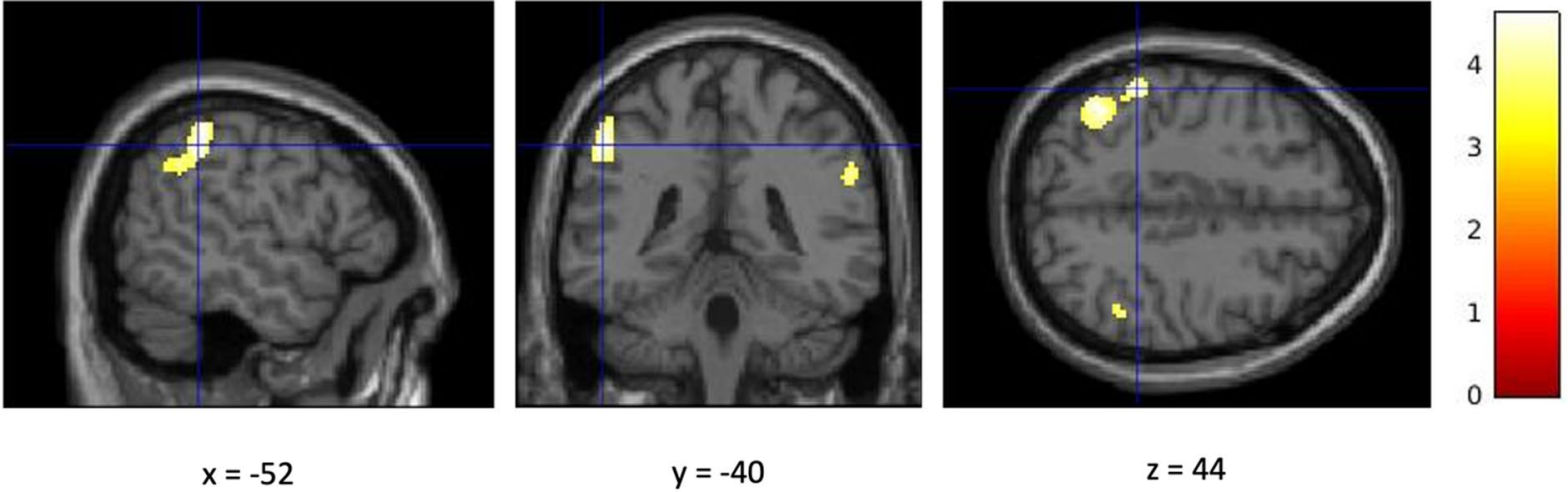
Cluster activation of the left supramarginal gyrus.

**Fig. 3:**
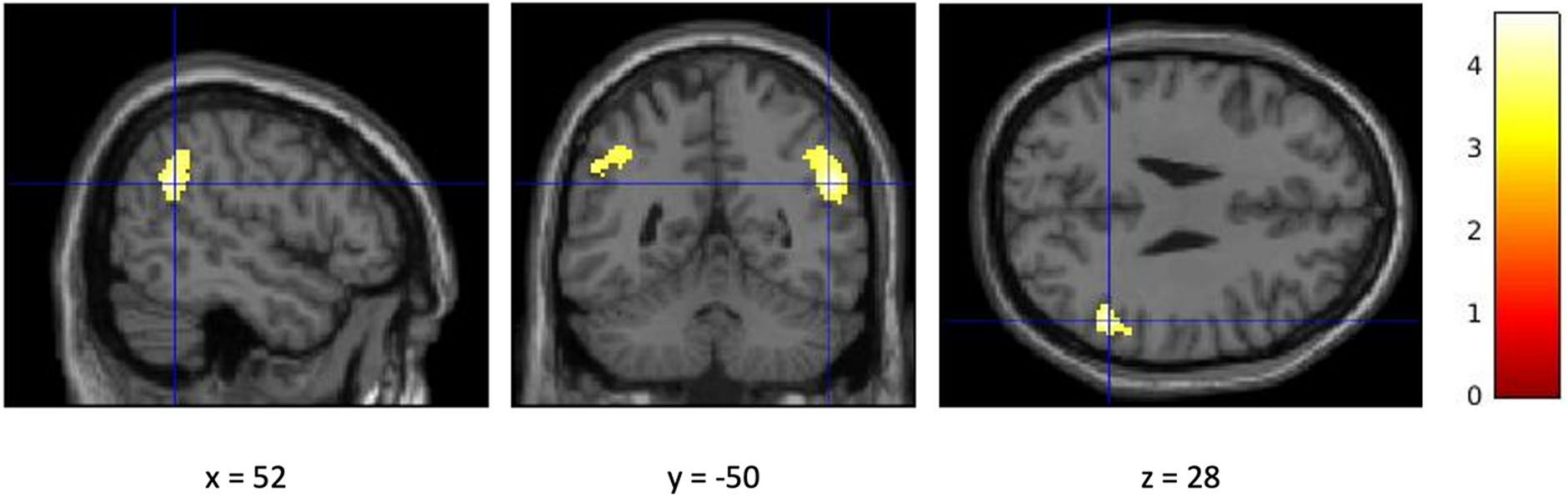
Cluster activation of the right angular gyrus.

A whole brain analysis examined contrasts between anodal and sham HD-tDCS across different task phases, including the decision, provocation, and outcome phases. In addition, parametric modulators for the decision and provocation phases were included. No significant differences in brain activation between anodal and sham HD-tDCS were observed for the decision, provocation, or outcome phases, nor for the parametric modulation of the decision phase (*p*_FWE_ > 0.05). The parametric modulation of provocation revealed that increasing levels of provocation were associated with greater activation in the left supramarginal gyrus (Fig. 2) and the right angular gyrus (Fig. 3) in the anodal HD-tDCS group compared to the sham condition (*p*_FWE_ < 0.05). Results are depicted in Table 5.

**Table 5:** Provocation parametric modulation.

Cluster	Region	L/*R*	x	y	z	k	t	*p*
Provocation phase; *parametric modulation* “provocation intensity”								
1	Supramarginal Gyrus (IPL/IPS)	L	-52	-40	44	490	4.60	0.003
2	Angular Gyrus (IPL)	R	52	-50	28	321	4.45	0.02

Contrast of activation clusters, p < 0.001 and cluster-level p (FWEcorrected) < 0.05. The coordinates are given according to the MNI space.

Figure 2 illustrates the parametric modulation of brain activation during the provocation phase by level of provocation (anodal > sham HD-tDCS). Cluster-level FWE corrected, *p* < 0.05 (at cluster *p* > 0.05 and cluster size of *k* = 490). The bar legend indicates the statistical significance.

Figure 3 illustrates the parametric modulation of brain activation during the provocation phase by level of provocation (anodal > sham HD-tDCS). Cluster-level FWE corrected, *p* < 0.05 (at cluster *p* > 0.05 and cluster size of *k* = 321). The bar legend indicates the statistical significance.

## Discussion

We observed a successful provocation of the TAP which aligns with previous research showing that provocation had a robust effect^3–5,34,35^. However, winning or losing the reaction time task did not influence subsequent punishment selections, unlike in studies using monetary manipulations^5^. Anodal HD-tDCS applied over the rIFG appeared to attenuate the effect of provocation on aggression. Although no direct main effect of anodal HD-tDCS on aggression was observed, stimulation of the rIFG was associated with a subtle but meaningful modulation of responsiveness to provocation. This behavioral pattern was paralleled by neural findings from the parametric modulation analysis, which revealed stronger activation in the left supramarginal gyrus and right angular gyrus with increasing provocation in the anodal HD-tDCS group. Considered jointly, the behavioral and neural results suggest that stimulation-related differences in brain activation may underlie the observed behavioral changes. Taken together, the present findings indicate that HD-tDCS targeting the rIFG may engage components of the inhibitory control network, which in turn are associated with a mitigation of the provocation effect on aggression. Notably, no significant behavioral effects were observed for decision or outcome phases, and corresponding whole-brain analyses for decision, decision-related parametric modulation, provocation, and outcome phases did not yield statistically significant results.

### Behavioral correlates

Our results revealed no overall reduction in aggression following anodal HD-tDCS compared to sham stimulation over the rIFG, consistent with prior tDCS research^22^. This absence of a main effect aligns with previous studies targeting the right DLPFC in healthy controls^5,35^, but contrasts with findings indicating that stimulation of the right VLPFC or right DLPFC can reduce aggression^19,36^. Importantly, meta-analytic evidence suggests that tDCS is not reliably associated with direct reductions in aggression when different stimulation montages are considered^13^.

Cumulative evidence suggests that the effects of tDCS are mixed and may be influenced by a range of moderating factors. Studies that fail to observe direct tDCS effects on aggression often report interaction effects with contextual or individual factors such as study design^24^, sex^15^, and genetic factors^35^. For instance, variation in the catechol-O-methyltransferase gene and sex differences have been shown to account for heterogeneity in tDCS effects on aggression-related outcomes^15,35^.

Although HD-tDCS did not produce a main effect on aggression, our findings revealed an interaction between HD-tDCS and provocation. Specifically, anodal HD-tDCS targeting the rIFG, compared to sham stimulation, was associated with attenuated responsiveness to provocation. This pattern suggests that HD-tDCS may have facilitated regulatory processes in situations requiring inhibitory control, thereby reducing provocation responsiveness. Notably, this interaction was detected using a trial-by-trial analytical approach, highlighting how aggression unfolded overtime and how tDCS may modulate sensitivity to provocation rather than overall aggression levels. Partly in line with our results, a previous tDCS study investigating right anodal/left cathodal and right cathodal/left anodal stimulation reported reduced aggression during high-provocation blocks. However, this effect was observed following left anodal stimulation^17^.

Contextualizing our findings is challenging, given the differences in study designs and the limited research applying HD-tDCS to reduce aggression. To our knowledge, only one study examined the influence of HD-tDCS on aggression. In a longitudinal design, HD-tDCS over the ventrolateral PFC, applied twice daily for five consecutive days, reduced aggression in the Point Subtraction Aggression Paradigm^32^. One common thread between our study and that of Sergiou et al. (2022)^32^ is that stimulation targets were located within the ventral PFC. Whereas our study targeted the lateral rIFG, Sergiou et al. (2022) applied stimulation to a more medial ventral PFC region. Despite differences in tasks and study designs, these converging findings highlight that HD-tDCS applied to ventral PFC regions may be beneficial for modulating aggression.

### Neural correlates

To our knowledge, this is the first study to examine the effects of HD-tDCS on TAP performance during fMRI, which limits comparisons with other studies. Our imaging results showed no differences in brain activation between anodal and sham HD-tDCS during the provocation phase. However, significant differences in brain activation between anodal and sham HD-tDCS emerged in the parametric modulation of the provocation phase. This aligns with our behavioral effects, indicating that the influence of HD-tDCS emerged with increasing provocation intensity. In the anodal HD-tDCS group, as compared to sham, increased provocation was associated with increased cluster activation in the right angular gyrus and left supramarginal gyrus.

Both regions are part of the inferior parietal cortex^37^ and the IPL^38^. Previous neuroimaging studies have highlighted the involvement of the IPL in provocation-related processes during the TAP^39^. Specifically, increased activation in the left IPL has been associated with higher as compared to lower provocation, whereas activation in the right IPL increased during high-provocation trials when punishment selections were low^39^. Moreover, heightened IPL activation has been linked to increased retaliatory behavior in a TAP version combining provocation and retaliation^34^.

Accordingly, the observed provocation-related activation in the IPL should not be interpreted as evidence for an isolated effect of rIFG stimulation, but rather as reflecting modulation of a broader fronto-parietal network influenced by HD-tDCS. Although stimulation was applied over the rIFG, its effects may not have been spatially confined to a single cortical locus and may have spread to functionally connected regions within the same network, as shown in previous work^40^.

The rIFG and IPL are functionally interconnected and are thought to jointly support inhibitory control and emotion regulation processes^29,41^. While the IPL may contribute to inhibitory control processes, prefrontal regions (e.g., IFG) are likely to play a more critical role during the initial stages of inhibition due to their direct involvement in the fronto-parietal pathway governing motor execution^28^. Given that both areas are considered core components of the inhibitory control network^28,41^, our findings may be interpreted as reflecting modulation of this broader network, as reflected by provocation-related increases in bilateral IPL activation. Notably, these activation differences between anodal HD-tDCS and sham conditions may correspond to the observed attenuation of provocation responsiveness. Hence, our findings support the notion that stimulation of the rIFG, alongside concomitant activation in the IPL, reflect engagement of an inhibitory control network that plays a critical role in the regulation of aggression^6,29^.

In contrast, other phases of the TAP, including the parametric modulation of decision and outcome, showed no differences in brain activation between anodal and sham HD-tDCS. While HD-tDCS did not produce a main effect on behavior and showed no significant differences in brain activation during the provocation or other task phases, the significant interaction between provocation and HD-tDCS, together with the corresponding neural effects revealed in the parametric modulation, indicates that behavioral effects and their neural correlates may aligned.

### Limitations

The findings of the current study must be considered in light of several limitations. First, the sample consisted exclusively of healthy male participants, which limits the generalizability of the results to female populations and to clinical groups. Healthy participants typically show intact inhibitory control and relatively low levels of aggression, potentially constraining the observable effects of neuromodulation. It is therefore conceivable that the effects observed here may be more pronounced in more vulnerable populations, such as patients with mental disorders or criminal offenders. However, the present findings cannot be generalized to females with respect to behavioral and neural outcomes. Based on previous work and the higher prevalence of aggressive and criminal behavior in men, the present study focused on a male cohort considered to be at increased risk for aggression^2^. Future studies should explicitly include female participants to examine potential sex-specific effects of HD-tDCS. Second, the relatively small sample size limits the generalizability of the findings. Nonetheless, given available resources and participant drop-out during fMRI acquisition, the sample size is comparable to that of previous tDCS studies investigating aggression-related outcomes (see, ^19^), and thus remains informative for the emerging literature.

Third, although our electrode montage was designed to primarily target the rIFG, we cannot exclude the possibility that the induced electric field extended into adjacent VLPFC regions. As such, stimulation effects may not have been entirely confined to the rIFG, which should be considered when interpreting the regional specificity of the neural findings and their clinical implications. Lastly, the TAP is a laboratory-based task performed in a highly controlled environment, which does not reflect real life aggressive behavior. Nevertheless, both the present behavioral findings and previous studies demonstrate that the TAP reliably elicits provocation aggressive responding^3–5,35^, supporting its validity as an experimental model of aggression.

## Conclusion

Building on previous research, the present findings indicate that anodal HD-tDCS targeting the rIFG mitigated effects of provocation on aggression. At the neural level, anodal HD-tDCS was associated with modulation of IPL activation compared to sham stimulation, potentially reflecting downstream effects of focal rIFG stimulation within a broader inhibitory control network. Behavioral and imaging findings in the present study may, to some extent, be explained by the enhanced regional specificity of HD-tDCS, which allowed for more focal stimulation of the rIFG - a region strongly associated with inhibitory control. This contrasts with the more diffuse electric field distribution typically produced by conventional tDCS, which might result in widespread and less specific stimulation effects^30,42^. In conclusion, these results suggest that HD-tDCS may influence provocation responsiveness by facilitating inhibitory control processes and could complement existing therapeutic approaches addressing emotion regulation. Nevertheless, future research will be pivotal in determining stimulation protocols with effective and replicable outcomes and to determine whether HD-tDCS can effectively modulate aggression in high-risk or clinical populations.

## Methods

### Participants

42 healthy male participants were recruited. One participant was excluded from the analysis due to a stimulation abortion after 10 min. The final sample consisted of 41 healthy, male, and right-handed participants (age = 28.76, SD = 9.83). 19 participants were in the anodal and 22 in the sham HD-tDCS group. The initial goal was to recruit two groups namely, healthy controls and criminal offenders. However, the study personnel experienced challenges with the offender recruitment. Due to drop-out only three offenders completed the entire study procedure. Hence, we excluded the three offenders and moved on with healthy controls until the end of the funding period. To determine appropriate sample sizes, we conducted an a priori power analysis (α = 0.05, power = 0.8) assuming an effect size of = 0.381, which suggested a sample size of 25 participants per group (active versus sham HD-tDCS). Due to sampling difficulties in the target population, this target could not be fully achieved; thus, the final sample represents a form of convenience sampling within feasible recruitment constraints^43^. We therefore conducted a post-hoc power analysis based on the interaction term in the final statistical model. The post-hoc power analysis was implemented in R using simr with fixed effects evaluated with Satterthwaite degrees of freedom^44^. A simulation-based post -hoc power analysis (1000 simulation, α = 0.05) for the interaction HD-tDCS x provocation yielded an estimated power of 95.9% (95% CI: 94.48–97.04) for the observed effects size (d = 0.06). Exclusion criteria consisted of common MRI and HD-tDCS contraindications, psychiatric and neurological diagnoses, history of head trauma and unconsciousness. All participants received monetary compensation of 50 euros after participation. This study was pre-registered at the German Clinical Trial Register (https://www.drks.de/search/de/trial/DRKS00028607/details).

### Design

This study was randomized, double-blind and sham controlled. To ensure balanced group sizes, participants were assigned to two stimulation protocols (anodal and sham HD-tDCS) using block randomization. To ensure blinding of the measuring personnel, four different protocols were created (A, B, C and D) with the Neuroelectrics Instrument Controller Software (NIC2.0) by the leading study personnel. Two protocols used identical anodal HD-tDCS settings and two other protocols identical sham settings. The measurement personnel and the participants were blind to this randomization.

### Procedure

Participants were invited to the laboratory located in the University Hospital RWTH Aachen. Written informed consent was obtained from all subjects following the initial briefing. To avoid socially desirable behavior during the task, we advertised the study to measure attention and concentration in a social and non-social context. Participants were told they would play against a real opponent in a competitive reaction time task. Each participant was randomly assigned to either the anodal or sham HD-tDCS group based on a predefined list. Afterwards, participants answered questionnaires including self-reports on impulsivity and aggression. Subsequently, a phone call was conducted to enhance the credibility of the opponent’s existence. Once participants were placed inside the MRI scanner, they performed the Stop Signal Task during HD-tDCS application. After termination of the stimulation, the HD-tDCS device was removed and participants reentered the scanner tunnel to complete the Taylor Aggression Paradigm (TAP). Active noise cancellation using the OptoACTIVE system (Optoacoustics Ltd, ST Mazoe Israel) was used to reduce the scanner noise. The MRI measurement was concluded with an anatomical scan. Post-session questionnaires to measure HD-tDCS side-effects and assess blinding were administered at the end of the study. The study procedure was approved by the ethics committee of the medical faculty of RWTH Aachen and conducted in accordance with the Declaration of Helsinki.

### HD-tDCS

**Fig. 4:**
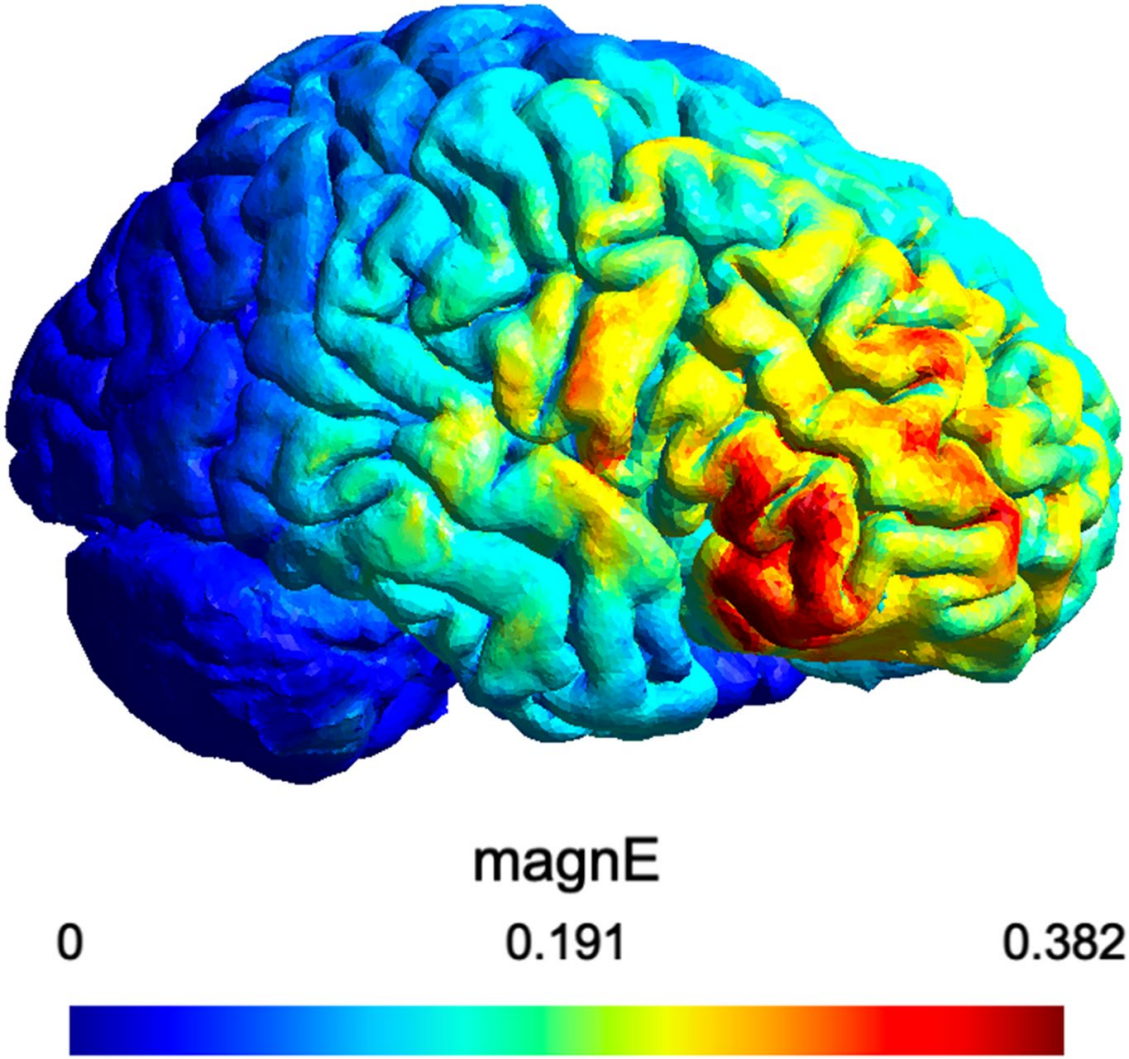
HD-tDCS electric field distribution.

Stimulation was delivered by the Starstim 8 system (Neuroelectrics, Barcelona, Spain) using the NIC2.0 software version number v2.0.6 (https://www.neuroelectrics.com/nic2). The HD-tDCS montage consisted of five 3.14 cm^2^ large electrodes. The anode was positioned at the F6 position of the 10–20 EEG system. Four reference electrodes were placed in a circular formation around the stimulation electrode with at least 8 cm distance to the target electrode F6. The following positions were used: TP8, PZ, FC3, and FP1. Stimulation was delivered at an intensity of 1.5 mA for a total duration of 21 min, including 30 s ramp-up and ramp-down periods. In the sham condition, participants received only the ramp-up and ramp-down phases (each 30 s), with no stimulation delivered in between. Following stimulation onset, the measurement personnel waited one minute before assessing participants’ well-being to minimize discomfort during HD-tDCS. The electric field distribution simulated with SimNIBS^45^ is depicted in Fig. 4.

Figure 4 shows the simulated electric field distribution of the HD-tDCS montage. The bar indicates the stimulation magnitude where the stimulation is the strongest (red) or weakest (blue). The simulation was calculated using SimNIBS.

### Neuropsychological assessment

Participants completed the self-reports of positive and negative affect administered before and after the MRI procedure. Other psychological tests involved the Reaction Proactive Aggression Questionnaire (RPQ)^46^, Aggression Questionnaire (AQ)^47^ and the Barrett Impulsivity Scale − 11 (BIS-11)^48^. A neuropsychological test battery including the Trial-Making Tests (TMT A/B)^49^ and a German vocabulary test (WST)^50^ were administered before the MRI procedure.

### Taylor Aggression Paradigm

**Fig. 5:**
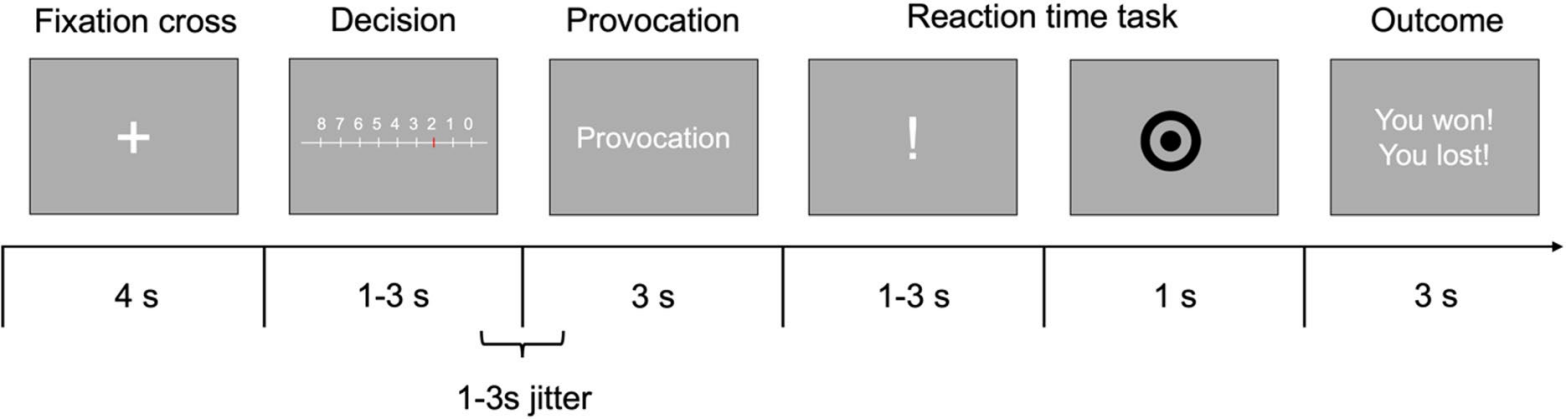
Taylor Aggression Paradigm. Shows a schematic overview of the Taylor Aggression Paradigm.

The TAP is a computerized task based on a competitive reaction time game^51^. This task requires a cover-story, where the deception of the participant ensures unadulterated measurement of socially undesirable behavior such as aggression. The task is divided into different phases, including punishment, provocation, a reaction-time task and an outcome phase. During the punishment phase, participants were asked to choose a punishment level for their opponent. Participants were also told that their opponent would get a punishment only if they lost the trial. The punishment involved a noise blast of 9 volume levels. Level 0 involved no noise, level 8 a maximum of 100 dB. An existing version of the TAP was modified by replacing monetary subtraction^4,5,35^ with the administration of noise blasts^52,53^. Exchanging the stimulus type from monetary to an auditory stimulus was done to minimize the risk for economic decision-making. In the provocation phase, the computer displayed the punishment level for the participant, presumably chosen by the opponent. The computer adjusted for extreme response styles, such as low punishment or high punishments by imitating the participant. Increasing provocation throughout the task was pre-defined. The reaction time task involved a jittered (1–3 s) exclamation mark to prepare the participant for the target stimulus, which required a button press upon presentation. Lastly, during the outcome phase, either a pleasant or aversive sound was presented. The win/lose ratio was equal by default; however, to increase believability of the cover story, a reaction time below (150 ms) or above (350 ms) a predefined time window resulted in a win or lose, respectively. If participants reacted faster than 150 ms they always won, while if they reacted slower than 350 ms they always lost. Each participant completed 75 trials, lasting approximately 25 min. The TAP procedure is illustrated in Fig. 5.

### Statistical analysis

#### Behavioral analysis

Behavioral analysis was conducted in R Studio Version 2024.04.2 + 764^54^. The TAP was analyzed in a trial-by-trial fashion adapted from previous studies^4,5,35^ aiming at increasing reproducibility. The first trial was deleted for the analysis because no provocation was introduced before that. A linear mixed model with random and fixed effect structures was estimated with the R package lme4 version 1.1.36 ^55^. The random intercepts and slopes consisted of trials nested within subject ID. This ensures that each participant is linked to all their trials. Fixed effects included provocation from the previous trial (0–8), outcome of the previous trial (win vs. lose) and HD-tDCS condition (anodal vs. sham), as well as an interaction term capturing the difference in slopes of provocation between HD-tDCS groups. The dependent variable was the punishment selection (0–8) in each trial. Post-hoc tests were conducted using emmeans version 1.10.6^56^, and simple slope comparisons for interaction effects were performed using the interactions package version 1.2.0^57^. To ensure convergence, the bobyqa function from the lme4 version 1.1.36^55^ package was used.

#### MRI acquisition

A 3T Siemens Prisma Scanner (Siemens AG; Erlangen, Germany) with a 20-channel head coil was used to acquire anatomical and functional images. Anatomical images were acquired with a TR of 2300ms, TE=298ms, flip angle = 9 degrees, voxel size = 1 × 1 × 1 mm, slice thickness = 1 mm, 34 number of slices and a matrix size of 256 × 256. Functional images were acquired with spin-echo EPI sequence with a TR of 2000ms, TE=28ms, flip angle = 77 degrees, voxel size = 1 × 1 × 1 mm, FOV = 256 × 256 mm, slice thickness = 3.3 mm, 34 number of slices and a matrix size of 64 × 64.

#### MRI analysis

FMRI data was analyzed using the SPM12 toolbox (Wellcome Department of Imaging Neuroscience, University College London, London, UK) in Matlab version 24.2 2024b^58^. Six participants were omitted from the analysis due to technical difficulties or excessive movements during the scan (larger movement or rotation than 5 mm). The fMRI analysis included 16 participants in the anodal and 19 participants in the sham group. The pre-processing pipeline included realignment, segmentation, co-registration, normalization, and smoothing with an 8 FWHM kernel. The analysis was adapted from previous research^35^. The time series of participants included four regressors of interest modelling the decision phase, the provocation phase, the outcome of won trials, and the outcome of lost trials. Other regressors of no interests were added, including anticipation of the reaction time game and the reaction time game. The model included one parametric modulator for the decision and one for the provocation phase. The first parametric modulator included the level of punishment (punishment selection). The second parametric modulator was the level of provocation (provocation intensity). Six realignment parameters were additionally used as regressors of no interest. The second level analysis included independent t-tests to compare mean values between anodal and sham HD-tDCS regarding the provocation, punishment and outcome phases. Two parametric modulators of the decision phase (“decision amount”) and the provocation phase (“provocation intensity”) were included. Regarding won > lost and lost > won trials, hrf regressors of the outcome phase were included. To ensure multiple comparison without family wise error rates a *p*(FWE) < 0.05 was used to correct cluster threshold for all analyses. Brain areas that exceeded the FWE Cluster threshold are reported. The functional clusters were anatomically localized with the Anatomy Toolbox^59^.

## Data Availability

The anonymized data can be shared from the corresponding author upon reasonable request.
